# MADGAN:A microbe-disease association prediction model based on generative adversarial networks

**DOI:** 10.3389/fmicb.2023.1159076

**Published:** 2023-03-23

**Authors:** Weixin Hu, Xiaoyu Yang, Lei Wang, Xianyou Zhu

**Affiliations:** ^1^College of Computer Science and Technology, Hengyang Normal University, Hengyang, China; ^2^Institute of Bioinformatics Complex Network Big Data, Changsha University, Changsha, China; ^3^Big Data Innovation and Entrepreneurship Education Center of Hunan Province, Changsha University, Changsha, China

**Keywords:** microbe-disease associations, graph convolution neural network, generative adversarial network, residual network, computational prediction model

## Abstract

Researches have demonstrated that microorganisms are indispensable for the nutrition transportation, growth and development of human bodies, and disorder and imbalance of microbiota may lead to the occurrence of diseases. Therefore, it is crucial to study relationships between microbes and diseases. In this manuscript, we proposed a novel prediction model named MADGAN to infer potential microbe-disease associations by combining biological information of microbes and diseases with the generative adversarial networks. To our knowledge, it is the first attempt to use the generative adversarial network to complete this important task. In MADGAN, we firstly constructed different features for microbes and diseases based on multiple similarity metrics. And then, we further adopted graph convolution neural network (GCN) to derive different features for microbes and diseases automatically. Finally, we trained MADGAN to identify latent microbe-disease associations by games between the generation network and the decision network. Especially, in order to prevent over-smoothing during the model training process, we introduced the cross-level weight distribution structure to enhance the depth of the network based on the idea of residual network. Moreover, in order to validate the performance of MADGAN, we conducted comprehensive experiments and case studies based on databases of HMDAD and Disbiome respectively, and experimental results demonstrated that MADGAN not only achieved satisfactory prediction performances, but also outperformed existing state-of-the-art prediction models.

## 1. Introduction

Microbes are far more numerous than human cells (Integrative HMP (iHMP) Research Network Consortium, 2014; Sender et al., 2016), and play an important role in human beings (Human Microbiome Project Consortium, 2012). The microorganisms parasitic on the human body constitute the human microbial community, and their composition varies from person to person (Human Microbiome Project Consortium, 2012). These microbial populations can not only protect the human body from foreign microorganisms and pathogens, but also participate in intestinal digestion and absorption, and promote metabolism (Guarner and Malagelada, 2003; Kau et al., 2011). Therefore, to some extent, the human microbial population can even be regarded as human “forgotten organs”(Quigley, 2013), the imbalance of microorganisms will not only lead to the occurrence of nervous system diseases, but also affect the immune and metabolic functions of the human body (Cenit et al., 2017; Li et al., 2017). For example, changes in intestinal microbiota are highly correlated with the pathogenesis of various nervous system diseases, including depression, autism (Kim et al., 2018), asthma (Al-Moamary et al., 2021) and cancer (Schwabe and Jobin, 2013), etc. Of course, there is also evidence showing that microbial populations can help regulate disease as well (Cryan and Dinan, 2012). For instance, researches show that lactic acid bacteria and bifid bacteria play a positive role in regulating anxiety, cognition, pain and depression symptoms (Desbonnet et al., 2010). In addition, Huang pointed out that microorganisms can affect the hypersensitivity and asthma of susceptible people. Early intervention to promote the healthy composition of human microbiome may help prevent asthma (Huang, 2013). Hence, it is meaningful to infer potential relationships between microorganisms and diseases, which can not only help researchers understand the pathogenesis of diseases, but also help us to prevent, diagnose and treat diseases, thus promoting global human health. Utilizing biotechnology to identify microbe-disease associations is time-consuming, costly and blind, so it is meaningful to identify potential microbe-disease associations through computational methods. Up to now, representative calculative methods can be roughly divided into four categories, such as the network-based, binary local features-based, matrix factorization/completion-based and graph neural network-based methods. Among them, the network-based methods infer latent microbe-disease associations by mainly adopting the topology information of different networks. For example, Chen et al. (2017) proposed a KATZ-based model KATZHMDA to infer possible microbe-disease associations based on a newly constructed heterogeneous network, which scores potential disease related microbes by step size and path numbers. Zeng et al. (2022) introduced the knowledge graph into the field of drug discovery, integrated data information through a displayed structure, and strengthened the structured connection and semantic relationship between entities. However, the methods based on binary local features focus on taking microbes and diseases as local objects, and identify potential microbe-disease associations by combining the features between them. For instance, Huang et al. (2017) developed a combined recommendation algorithm based on neighborhood and graph by integrating two independent recommendation models to recommend disease related microbes. In addition, Matrix factorization/completion-based methods aim to decompose the known incidence matrix into two characteristic matrices, and approximate the incidence matrix with the product of the two matrices. For instance, Shen et al. (2017) proposed a matrix factorization-based model for microbe-disease association prediction, which integrated known microbe-disease associations and introduced a collaborative matrix factorization scheme to update the correlation matrix about microbes and diseases for inferring the most possible disease-related microbes. Finally, the graph neural network-based methods used to learn structural data by taking microbe and disease related data as the input of the neural networks, so as to extract and explore features and patterns in graph structural data. For example, Long et al. (2021) developed a graph attention network with inductive matrix completion to detect potential microbe-disease associations. Cheng et al. (2021) used the deep generative model as an entry point to discuss and study the *de novo* molecular design for drug discovery (*de novo* molecular design for drug discovery).

The emergence of generative adversarial networks is another milestone in the field of computer vision. It provides a new tool for solving various image prediction problems. For instance, in 2014, Lan et al. proposed a framework for estimating the generative adversarial network model through the confrontation process, and improved the ability of the model through the mutual game between generative adversarial networks (Goodfellow et al., 2020). However, the generative adversarial network still has problems such as unstable results and difficult training. Hence, Arjovsky et al. (2017) conducted a theoretical analysis of the generative adversarial network and provided an optimal solution. Later, new results appeared in the field of image processing, such as Style GAN (Karras et al., 2019), Cycle GAN (Zhu et al., 2017), SeCGAN (Wu et al., 2019), etc. In recent years, many researchers have begun to explore the application of generative adversarial networks in other fields. For example, Lei et al. (2019) applied it in the direction of dynamic information generation to build a nonlinear time link prediction model. Dai et al. (2021) introduced generative adversarial networks to natural language translation work. Zheng et al. (2022) utilized a generative adversarial network model to predict urban traffic flow.

In this paper, a generative adversarial network framework called MADGAN was designed for latent microbe-disease association prediction, in which, a GCN was adopted to obtain the microbe-disease association features first, and then, we would train the ability of MADGAN by games between the generation network and the decision network. And at the same time, inspired by the idea of residual network, we introduced the cross-level weight distribution structure to enhance the depth of the network to prevent over-smoothing during the model training process. Finally, intensive experiments based on the *k*-fold cross-validation framework were implemented to compare the prediction performance between MADGAN and state-of-the-art prediction models. And as a result, MADGAN was proved to be of satisfactory prediction ability and outperformed existing representative competing models.

## 2. Materials and methods

### 2.1. Construction of the microbe-disease association network

In this section, we would download known microbe-disease associations from two well-known public databases including HMDAD (Ma et al., 2017) and Disbiome (Janssens et al., 2018) respectively. Among them, HMDAD^1^ is the first microbe-disease association database constructed by ma et al. in 2017, which contains 483 known microbe-disease associations. After removing duplicate data, we finally obtained 450 different known microbe-disease associations between 39 diseases and 292 microbes. Besides, Disbiome^2^ is a public microbe-disease association database constructed by Janssens et al., in which, there are 5,573 known associations between 240 diseases and 1,098 microbes collected from published academic papers. After removing duplicate data, we finally derived 4,351 known microbe-disease associations between 218 diseases and 1,052 microbes. For convenience, let $n_{d}$ and $n_{m}$ denote the numbers of newly-downloaded diseases and microbes respectively, then we can obtain a adjacency matrix $A\in {\mathbb{R}}^{n_{d}\times n_{m}}$ as follows: for any given disease $d_{i}$ and a microbe $m_{j}$, if there is a known association between them, there is $A_{ij}$=1, otherwise, there is $A_{ij}$=0.

### 2.2. Multiple similarity calculation of disease

#### 2.2.1. Gaussian interaction profile kernel similarity of disease

Based on the assumption that two similar diseases will show similar interaction and non-interaction relationship with the same microorganism (Chen et al., 2017), in this section, we will first calculate the Gaussian interaction profile kernel similarity between a pair of diseases $d_{i}$ and $d_{j}$ as follows:


(1) $$GD\left(d_{i},d_{j}\right)=\exp\left(-\lambda_{d}\|A\left(i,:\right)-A\left(j,:\right){\|}^{2}\right)$$

Where A(i,:) and A(j,:) represent the $i^{th}$ and $j^{th}$ rows of the adjacency matrix A respectively, and $\lambda_{d}$ denotes the normalized kernel bandwidths that can be calculated as follows:


(2) $$\lambda_{d}=\frac{1}{\left(\frac{1}{n_{d}}\sum_{i=1}^{n_{d}}\|A\left(i,:\right){\|}^{2}\right)}$$

#### 2.2.2. Cosine similarity of disease

Based on the assumption that if two diseases are similar to each other, then their cosine curves will be more coincident, in this section, we will define the cosine similarity between a pair of diseases $d_{i}$ and $d_{j}$ as follows:


(3) $$CD\left(d_{i},d_{j}\right)=\left(\;A\left(i,:\right)\cdot A\left(j,:\right)\right)/\left(\left|A\left(i,:\right)|*|A\left(j,:\right)\right|\right)$$

The result of cosine similarity has good stability and certainty, the calculation speed is fast and the result is more intuitive. Suitable for large-scale information retrieval. Where A(i,:)⋅A(j,:) denotes multiplying the vectors of row i and row j, |A(i,:)| represents the mode of A(i,:), and |A(j,:)| represents the mode of A(j,:). |A(i,:)|∗|A(j,:)| represents the multiplication of two moduli, and then the value of the modulus is removed by the product of the vector, and finally the cosine value of the angle between the two diseases is obtained, that is, the cosine similarity. The calculation result of cosine similarity is between −1 and 1. When the similarity between two diseases is extremely high, the calculation result tends to be 1. When the similarity between two diseases is very low, the calculation result tends to −1.

#### 2.2.3. Functional similarity of disease

Based on the assumption that similar diseases tend to interact with similar genes, in this section, we will calculate the disease functional similarity based on the functional associations between disease-related genes (Xu and Li, 2006; Wei and Liu, 2020) as follows: Firstly, we download the gene interactions from HumanNet database^3^, in which, every interaction has an associated log-likelihood score (LLS). And then, for any given diseases $d_{i}$ and $d_{j}$, let $G_{i}=\left\{g_{i_{1}},g_{i_{2}},\ldots ,g_{i_{m}}\right\}$ and $G_{j}=\left\{g_{j_{1}},g_{j_{2}},\ldots ,g_{j_{n}}\right\}$ denote the newly-obtained gene sets of $d_{i}$ and $d_{j}$ separately, we will define the functional similarity between $d_{i}$ and $d_{j}$ as follows:


(4) $$DFS\left(d_{i},d_{j}\right)=\frac{\sum_{g_{k}\in G_{i}}F_{G_{j}}\left(g_{k}\right)+\sum_{g_{k}\in G_{j}}F_{G_{i}}\left(g_{k}\right)}{m+n}$$

Where $F_{G_{t}}\left(g_{p}\right)=\underset{g_{q}\in G_{t}}{\max}\left(FSS\left(g_{p},g_{q}\right)\right)$, and $FSS\left(g_{p},g_{q}\right)$ is the functional similarity score between the genes $g_{p}$ and $g_{q}$, which can be calculated as follows:


(5) $$FSS\left(g_{p},g_{q}\right)=\{\begin{array}{l} \;\;\;\;\;\;\;\;\;\;\;\;\;\;\;\;\;\;\;\;\;\;\;1\;\;\;\;\;\;\;\;\;\;\;\;\;\;if\;p=q\\ \frac{LLS\left(g_{p},g_{q}\right)-LLS_{\min}}{LLS_{\max}-LLS_{\min}}\;\;\;\;\;\;\;if\;p\neq q \end{array}$$

Where $LLS_{\max}$ and $LLS_{\min}$ represent the maximum value of *LLS* and the minimum value of *LLS* in HumanNet, respectively.

Thereafter, by combining above GIP kernel similarity, disease cosine similarity and functional similarity of disease, we can obtain an integrated similarity matrix of disease as follows:


(6) $$DS=\frac{GD+CD+DFS}{3}$$

### 2.3. Multiple similarity calculation of microbe

#### 2.3.1. Gaussian interaction profile kernel similarity of microbe

In the same way, we can calculate the gaussian interaction profile kernel similarity between any two microbes $m_{i}$ and $m_{j}$ as follows:


(7) $$MD\left(m_{i},m_{j}\right)=\exp\left(-\lambda_{m}\|A\left(:,i\right)-A\left(:,j\right){\|}^{2}\right)$$

Where A(:,i) and A(:,j) represent the $i^{th}$ and $j^{th}$ columns of the adjacency matrix A respectively, and $\lambda_{m}$ denotes the normalized kernel bandwidths that can be calculated as follows:


(8) $$\begin{array}{c} \lambda_{m}=\frac{1}{\left(\frac{1}{n_{m}}\sum_{i=1}^{n_{m}}\|A\left(:,i\right){\|}^{2}\right)} \end{array}$$

#### 2.3.2. Cosine similarity of microbe

Similarly, the cosine similarity between any two microbes $m_{i}$ and $m_{j}$ can be obtained as follows:


(9) $$CM\left(m_{i},m_{j}\right)=\;\left(A\left(:,i\right)\cdot A\left(:,j\right)\right)/\left(\left|A\left(:,i\right)|\times |A\left(:,j\right)\right|\right)$$

The calculation process of cosine similarity between two microorganisms is the same as that of disease cosine similarity. Similarly, when the similarity between two microorganisms is extremely high, the calculation result tends to be 1. When the similarity between two microorganisms is very low, the calculation result tends to −1.

#### 2.3.3. Functional similarity of microbe

In this section, we will calculate the functional similarity of microbe by using the following method proposed in the reference (Zhang et al., 2018): for any given disease $d_{t}$, it is first represented by a Directed Acyclic Graph $DAG_{d_{t}}=\left(V_{d_{t}},E_{d_{t}}\right)$, where $V_{d_{t}}$ includes the disease $d_{t}$ and its ancestor diseases, $E_{d_{t}}$ contains all the directed edges from parent nodes to children nodes (Wang et al., 2010), and then, the semantic contribution of the disease $d_{l}$ in $V_{d_{t}}$ to $d_{t}$ is defined as:


(10) $$\begin{array}{l} SC_{d_{t}}\left(d_{i}\right)=\\ \;\;\;\;\;\;\;\;\;\;\;\{\begin{array}{l} \;\;\;\;\;\;\;\;\;\;\;\;\;\;\;\;\;\;\;\;\;\;\;\;\;\;\;\;\;\;1\;\;\;\;\;\;\;\;\;\;\;\;\;\;\;\;\;\;\;\;\;\;\;\;\;\;\;\;\;\;\;\;\;\;\;\;\;\;\;\;\;\;\;\;\;if\;d_{l}=d_{t}\;\\ \max\left\{0.5\times SC_{d_{t}}\left(d_{l}^{\prime }\right)|d_{l}^{\prime }\in childrenof\;d_{l}\right\}\;\;\;\;\;otherwise\; \end{array} \end{array}$$

The semantic value of disease $d_{t}$ is formulated by:


(11) $$SV_{d_{t}}=\underset{d_{l}\in V_{d_{t}}}{\sum }SC_{d_{t}}\left(d_{l}\right)$$

Then, the semantic similarity between any two diseases $d_{i}$ and $d_{j}$ can be defined as follows:


(12) $$DSS\left(d_{i},d_{j}\right)=\frac{\sum_{d_{l}\in V_{d_{i}}\cap V_{d_{j}}}\left(SC_{d_{i}}\left(d_{l}\right)+SC_{d_{j}}\left(d_{l}\right)\right)}{SV_{d_{i}}+SV_{d_{j}}}$$

Besides, based on above formulae, we can further define the similarity between the disease $d_{i}$ and a set of diseases *D* as follows:


(13) $$DS\left(d_{i},D\right)=\underset{d_{j}\in D}{\max}\left(DSS\left(d_{i},d_{j}\right)\right)$$

Hence, for any two given microbes $m_{i}$ and $m_{j}$, we can calculate the function similarity between them as follows:


(14) $$MFS\left(m_{i},m_{j}\right)=\frac{\sum_{d_{j}\in D_{j}}DS\left(d_{j},D_{i}\right)+\sum_{d_{j}\in D_{i}}DS\left(d_{j},D_{j}\right)}{\left|D_{i}\right|+\left|D_{j}\right|}$$

Where $D_{i}$ denotes the set of diseases associated with the microbe $m_{i}$, and $D_{j}$ represents the set of diseases associated with the microbe $m_{j}$.

Obviously, by combining above GIP kernel similarity, disease cosine similarity and functional similarity of microbe, we can obtain an integrated similarity matrix of microbe as follows:


(15) $$MS=\frac{MD+CM+MFS}{3}$$

### 2.4. Construction of the heterogeneous network

Based on above descriptions, it is easy to see that we can construct a heterogeneous network Y through integrating the integrated similarity matrix DS of disease and the integrated similarity matrix MS of microbe with the adjacency matrix *A* as follows:


(16) $$Y=\left[\begin{array}{cc} DS & A\\ A^{T} & MS \end{array}\right]$$

## 3. Methods

**Figure 1:**
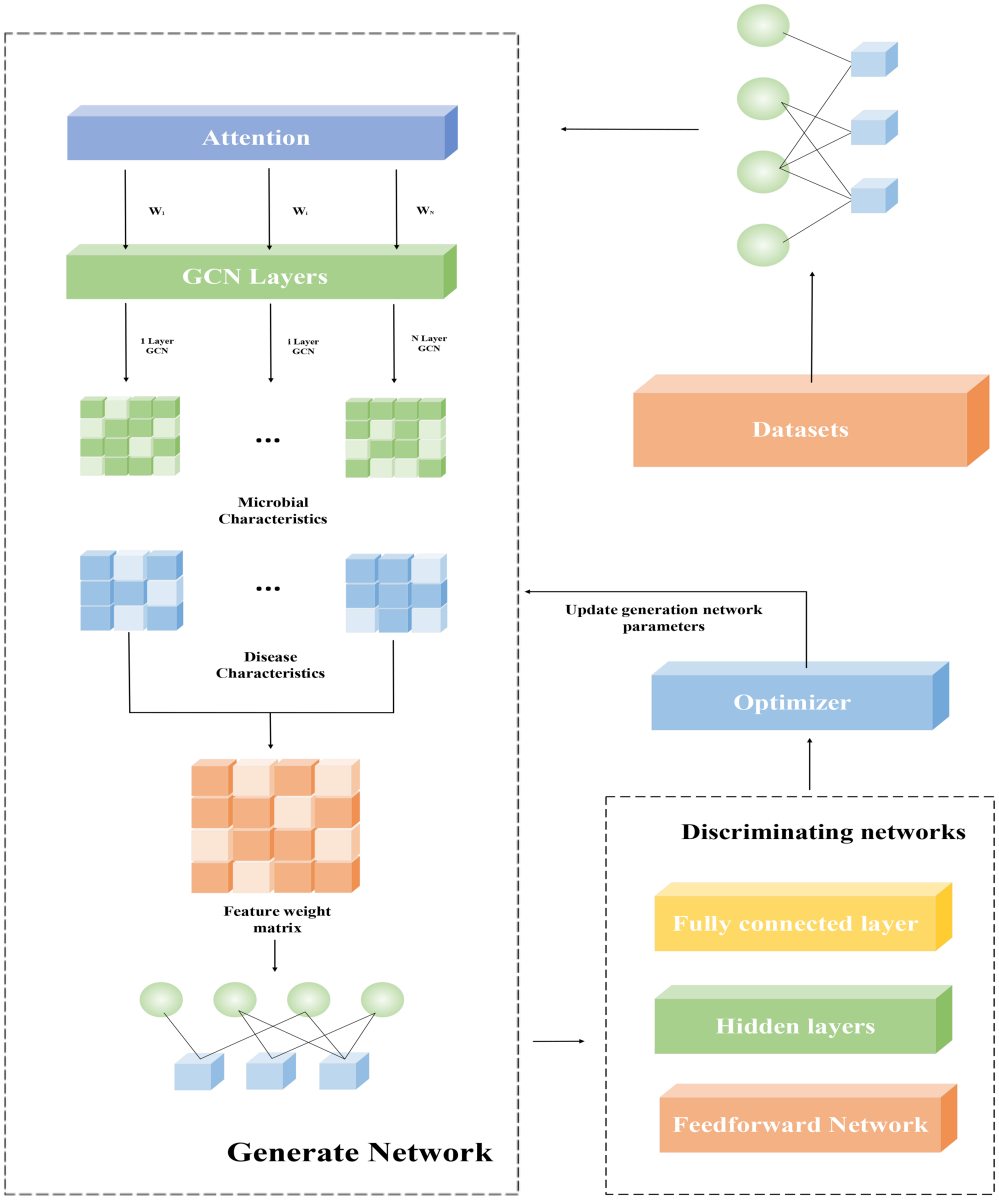
The general framework of the model.

The main framework of this paper is generative adversarial networks. A generative adversarial network consists of a generative network and a decision network, and it works by enhancing the model’s capabilities during the mutual gaming of the two networks. As shown in Figure 1, the information of known microbial-disease association data is extracted from the database, and after the calculation of similarity, it is input into the generative network. The core of the generative network consists of a GCN layer and an attention mechanism, which consists of a graph convolutional layer and a sparse graph convolutional layer. The data are passed through the generative network to generate prediction results, and the prediction results and the original sample data are input into the discriminator, which distinguishes the real results from the generated results and returns to update the model parameters of the generative network. This is a game process, in which the generative network needs to generate prediction results that are sufficient to confuse the judgment of the discriminator, while the discriminator needs to correctly distinguish the generated results from the true results. The ability of the generative network model is continuously improved during the game until the discriminator and the generative network reach an equilibrium, i.e., the probability of both the predicted and true outcomes is one half.

The generator network uses the information of the data set to output data samples, and the generator G(•) obtains a random sample z from the data samples, and z conforms to the p(z) probability distribution. After the generator generates data, it will be sent to the discriminator D(•), and the discriminator will try to predict the authenticity of the data after receiving real data or generated data. At the same time, it also needs a sample x from the real data distribution $p_{data}\left(x\right)$, the discriminator uses the activation function to solve a binary classification task, and outputs a value of 0–1 to distinguish the real result from the predicted result.

The game process of generative adversarial networks can be expressed as follows:


(17) $$\begin{array}{l} \min\;\max\;V\left(D,G\right)=\\ \;\;\;\;\;\;\;\;\;\;E_{x\sim p_{data}\left(x\right)}\left[logD\left(x\right)\right]+E_{z\sim p\left(z\right)}\left[1-logD\left(G\left(z\right)\right)\right] \end{array}$$

Among them, x is the real feature matrix, and G(z) is the feature matrix generated by the generation network. $p_{data}\left(x\right)$ is the probability distribution of x, and p(z) is the probability distribution of z. The optimization goal of training D to adjust its parameters is to maximize D(x) and minimize D(G(x)), and the optimization goal of training G to adjust its parameters is to minimize maxV(D,G). E stands for entropy, $x\sim p_{data}\left(x\right)$ stands for x is from $p_{data}\left(x\right)$ real data distribution. The meaning represented by $E_{x\sim p_{data}\left(x\right)}\left[logD\left(x\right)\right]$ is the entropy value from the real data distribution after passing the identifier. For data from the real data distribution, the ideal goal of the discriminator is to fully identify it, that is, predict the result as 1. Therefore, $E_{x\sim p_{data}\left(x\right)}\left[logD\left(x\right)\right]$ can also be regarded as the probability of the discriminator to distinguish real data, and the higher the probability, the better. The log function does not affect the relationship between variables, and its function is to amplify our loss to facilitate the calculation and optimization of the model. $E_{z\sim p\left(z\right)}\left[1-logD\left(G\left(z\right)\right)\right]$ can be regarded as the entropy value after the input generated data passes through the discriminator, and also represents the probability of the discriminator to distinguish the fake sample data. The smaller the probability, the better. minmaxV(D,G) is expressed as a confrontation between the generator and the discriminator. The generator G(•) hopes that the discriminator cannot distinguish fake samples, so it hopes to minimize the result of 1−logD(G(z)). The discriminator is the opposite, it hopes to better distinguish between true and false, that is, the result of maximizing 1−logD(G(z)). This is also the origin of this formula. At the end of training, there will often be a balanced form.

**Figure 2:**
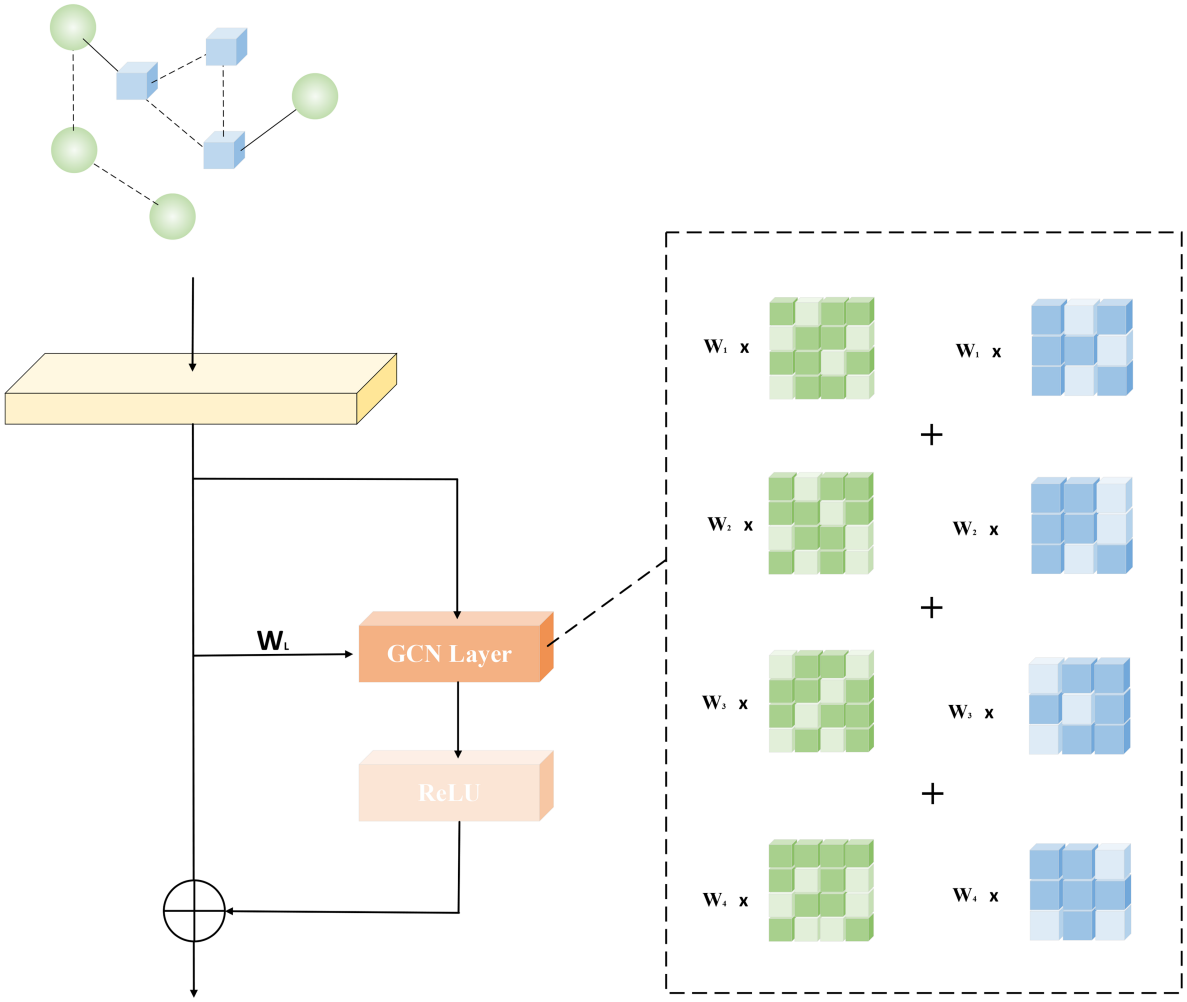
Generate network core model structure diagram.

The core of the principle of generative adversarial networks lies in the game between the generative network and the decision network. The core of the generative network is composed of GCN layers. In order to deepen the model depth of the generative network and thus generate more accurate prediction results, we use a residual network-like idea to optimize the model. We deepen the network while retaining the shallow features according to the weights, which makes the model less susceptible to phenomena such as oversmoothing and gradient explosion during the iterative process. As shown in Figure 2, the direct mapping is shown on the left, and the associated graph convolution operation and activation function are shown on the right.

The purpose of adding this structure is to increase the depth of the network. Under this premise, problems such as over-smoothing and gradient explosion are avoided. At the same time, combined with the attention mechanism, we have carried out weight ratios on both sides on the basis of similar residual ideas to achieve better results. Its formula derivation is as follows:


(18) $$h_{l}=h_{0}+\sum_{i,j=1}^{L}F\left(h_{i},W_{j}\right)$$

Among them, $h_{L}$ is the feature matrix output by each layer, and l∈{1,..,L}. $W_{j}$ is the weight assigned to each layer, and F(•) is the graph convolution function.

And the relevant formula of F(•) is as follows:


(19) $$F{\left(z,W\right)}_{l}=f\left(F{\left(z\right)}_{l-1},Y\right)=\mu \left(D^{-\frac{1}{2}}YD^{-\frac{1}{2}}F{\left(z\right)}_{l-1}W_{l-1}\right)\;$$

Where l∈{1,..,L}, $F{\left(z\right)}_{l}$ is the feature matrix generated by the lth layer GCN network, $D=diag\left(\sum_{j=1}^{N_{m}+N_{d}}Y_{i,j}\right)$ is a diagonal matrix, and $W_{l}$ is the weight matrix trained on the lth layer. And μ(•) is an activation function. In this paper, the RELU function is used as the activation function. The formula is as follows:


(20) $$RELU\left(x\right)=\{\begin{array}{c} \;x,\;x>0\\ \;0,x\leq 0 \end{array}$$

The weight calculation formula of $W_{l}$ is as follows:


(21) $$W_{l}=\frac{1}{L}$$

Graph Convolution (GCN) is a convolutional model applied by CNN in the field of graph structure. Different from CNN to achieve feature extraction by processing pixels, graph convolution uses spectral graph theory to map the graph structure transformation to the frequency domain through Fourier transform for processing, and finally perform inverse transformation. Compared with CNN that handles neat pixels, GCN can more effectively extract the correlation features between two points. For data with associated structures, the ability to effectively extract spatial features brought by GCN can better help them complete their tasks. In our model, the reconstructed heterogeneous network feature matrix is input into the generative network and processed as the input of the GCN model. Formula (19) reflects the training process of the GCN model, and z is the input data. The function of $D^{-\frac{1}{2}}{\text{YD}}^{-\frac{1}{2}}$ is to dilute the importance of nodes with high degrees, and to balance the weight information of nodes with different degrees. Therefore, formula (19) can also be simplified as:


(22) $$F{\left(z,W\right)}_{l}=\mu \left(\tilde{Y}F{\left(z\right)}_{l-1}W_{l-1}\right)\;$$

Among them, the role of $\tilde{Y}F{\left(z\right)}_{l-1}$ is to retain the information inherited by the upper layer nodes during the information transmission process, that is, to aggregate the information of the surrounding nodes to update the information of its own nodes.

The role of the discriminator is to distinguish between real and fake samples, and our discriminator consists of a fully connected feed-forward network, a hidden layer and an output layer. The discriminator alternately receives generated samples and real samples, and updates the parameters of the generated network through the discriminative results. Here we adopt the framework of WassersteinGAN to train the discriminator. The biggest difference between WGAN and traditional GAN is that the output layer is a linear layer and does not require a nonlinear activation function. Expressed in a formula it is:


(23) $$D\left(z\right)=\mu \left(z^{\prime }W_{h}+b_{h}\right)\;W_{o}+b_{o}$$

Among them, z is the input data, and z is the long vector after dimension reconstruction. μ(•) is the activation function of the hidden layer, $W_{h}$ and $b_{h}$ are the hidden layer parameters, and $W_{o}$ and $b_{o}$ are the output layer parameters.

As shown in Algorithm 1, the input is a known microbial-disease association matrix A. The similarity matrix of microorganisms and diseases is computed to construct the heterogeneous network Y. The new feature matrix is fed into the generative network. After initializing the optimizer, the generated prediction results are output after N rounds of training. The generated prediction results and sample data are input into the discriminator, and the parameter information of the generative network is updated according to the output results of the discriminator, and the completed generative network model is saved after several rounds of training.

**Table:** 

Algorithm 1: Algorithm of our proposed method
Inputs: Known associations matrix $A\in {\mathbb{R}}^{n_{m}\times n_{d}}$, microbe similarity matrix $K_{s}^{m}\in {\mathbb{R}}^{N_{m}\times N_{m}}$, disease similarity matrix $K_{s}^{d}\in {\mathbb{R}}^{n_{d}\times n_{d}}$; Output: The completed training of the generative network model Step 1: Constructing the heterogeneous network $Y\in {\mathbb{R}}^{\left(n_{m}+n_{d}\right)\times \left(n_{d}+n_{m}\right)}$ according to Formula (16); Step 2: Input the feature matrix into the generative network, initializing Optimizer Parameter Information; Step 3: for i=1→Ndo (*N* is the number of training rounds of the generative adversarial network) for l=1→Ldo (*L* is the depth of the graph convolution model) Compute the feature embedding of the *L* layer and output the generated prediction results end for Input the generated results and sample data into the decision network Update optimizer parameter information
end for Step 4: Save the model of the generative network

## 4. Experiments and results

### 4.1. Experimental setup

In this section, we adopted 5-fold cross validation(5cv) and 2-fold cross validation to assess the performance of our model. In the k-fold cross validation framework, all known microbe-disease associations in HMDAD and Disbiome were divided to *k*-subsets. In the process of model training, (*k*-1)-subsets are selected as the training set, and the remaining one as the test set. It is worth noting that there are no known negative samples, we regarded unknown associations as negative samples. After the training samples are input into MADGAN, all association pairs will get a predictive value. If the prediction score is higher than the given threshold, it will be considered as successful prediction. Obviously, different true positive rate and false positive rate can be obtained when setting different thresholds. The specific calculation formula is as follows:


$$\text{TPR}=\frac{TP}{TP+FN}$$

(24) $$\text{FPR}=\frac{FP}{FP+TN}$$

Where TP and TN represent the numbers of positive samples correctly judged as positive samples and negative samples correctly judged as negative samples, respectively; FP and FN are the numbers of negative samples incorrectly judged as positive samples and positive samples incorrectly judged as negative samples. By setting different thresholds, we can get multiple groups of different TPRs and FPRs. Then, TPR and FPR under different thresholds are taken as the x-axis and y-axis respectively, the receiver operating characteristics (ROC) can be further plotted, and the area under the line is taken to evaluate the prediction performance of the model.

### 4.2. Parameter analysis

**Figure 3:**
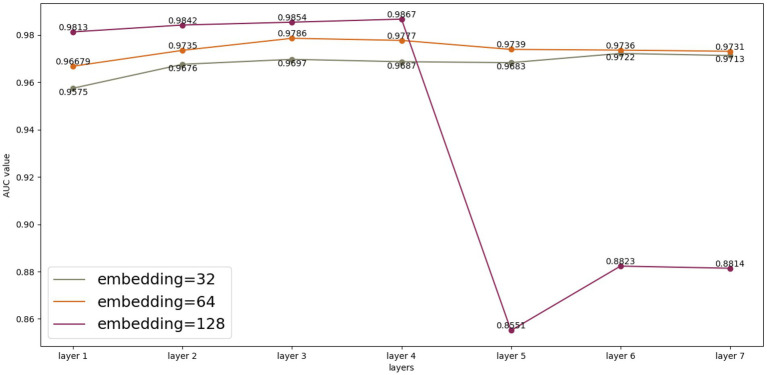
Model parameters analysis on the HMDAD dataset.

We performed multiple experimental and parametric analyses on the HMDAD database and the Disbiome database, respectively. As shown in Figure 3, we analyzed the experimental results generated by HMDAD in terms of the number of layers and embedding. We used a similar idea of residual network to deepen the number of layers of GCN to 4. After several rounds of training, the experimental results and loss values were maintained at a certain level, but we could see from the experimental results that after the number of layers was raised to 5, the experimental results could not be maintained at a certain level as in the previous layers, which we judged to be due to the limitation of the size of the dataset that made it impossible to deepen the network further. We judge that this is due to the limitation of the dataset size, which makes it impossible to deepen the network further, otherwise the phenomenon of oversmoothing will occur. We also compared different embedding values. Different embedding values take different time to train. When the embedding value is 128, the training time cost is greater than when the embedding value is 32. However, when the model depth is deepened to 5 layers, the embedding value of 128 cannot maintain good experimental results, and the embedding values of 32 and 64 are not affected much, but we think that further deepening the model depth and embedding values of 32 and 64 is also oversmoothing can occur, resulting in poor results.

For the Disbiome database, we also conducted multiple experiments, but the Disbiome database is much larger than the HMDAD database, and we were able to maintain the results at a certain level after deepening the GCN layers with our network up to 20 layers, without reaching the limit. We did not find the limit value due to the limitation of the experimental equipment, but we can understand that the experimental results did not deteriorate after deepening to more than 20 layers.

### 4.3. Comparison with state-of-the-art methods

In order to evaluate the performance of MADGAN, we compare our model with six state-of-the-art methods that includes network-based methods, binary local features-based methods, matrix factorization/completion-based methods and graph neural network-based methods. KATZHMDA and NTSHMDA are network-based methods, NGRHMDA and BiRWMP are binary local features-based methods, GRNMFHMDA is matrix factorization-based method, and GATMDA is graph neural network-based method. The comparison results of all these methods were shown in Tables 1, 2 respectively.

**Table 1:** Comparison performance between our model and state-of-the-art models based on HMDAD dataset.

Methods	AUC(5-fold cv)	AUC(2-fold cv)
KATZHMDA (Zhu et al., 2021) (network-based)	0.8703±0.0199	0.8755±0.0103
NTSHMDA (Luo and Long, 2018) (network-based)	0.8982±0.0312	0.8615±0.0151
NGRHMDA (Huang et al., 2017) (binary local features-based)	0.8921±0.0327	0.8929±0.0059
BiRWMP (Luo and Xiao, 2017) (binary local features-based)	0.8777±0.0089	0.8698±0.0079
GRNMFHMDA (He et al., 2018) (matrix factorization-based)	0.8806±0.0156	0.8756±0.0164
GATMDA (Long et al., 2021) (graph neural network-based)	0.9554±0.0184	0.9538±0.0049
Our model	0.9867±0.0078	0.9708±0.0117

**Table 2:** Comparison performance between our model and state-of-the-art models based on Disbiome dataset.

Methods	AUC(5-fold cv)	AUC(2-fold cv)
KATZHMDA (Zhu et al., 2021) (network-based)	0.6779±0.0141	0.6696±0.0058
NTSHMDA (Luo and Long, 2018) (network-based)	0.8294±0.0071	0.8086±0.0058
NGRHMDA (Huang et al., 2017) (binary local features-based)	0.8313±0.0052	0.8233±0.0046
BiRWMP (Luo and Xiao, 2017) (binary local features-based)	0.8344±0.0089	0.8139±0.0060
GRNMFHMDA (He et al., 2018) (matrix factorization-based)	0.8609±0.0047	0.8501±0.0017
GATMDA (Long et al., 2021) (graph neural network-based)	0.9307±0.0079	0.9296±0.0154
Our model	0.9428±0.0026	0.9290±0.0068

As shown in Tables 1, 2, we used 5 times of cross-validation and 2 times of cross-validation to conduct comparative experiments on the two databases. In experiments on the HMDAD database, our model performs better than other models. The 5-fold cross-validation method makes better use of the data set than the 2-fold cross-validation method, so it performs better. The data sample size of the Disbiome database is much larger than that of HMDAD, and its training time is also much longer than that of HMDAD. However, compared with HMDAD, the experimental results of all models have declined. We believe that part of the reason is that the depth of the model cannot support the training of a large number of sample data. Even if we use the method to deepen the depth of the model, it can only slightly improve the experimental effect. Another part of the reason may be because of the equipment environment.

## 5. Case study

In this section, we choose three diseases of asthma, Chronic Obstructive Pulmonary Disease (COPD) and Type 2 Diabetes (T2D) for case studies on the HMDAD to further verify the performance of our model. Specifically, we rank the above three related microorganisms in the predicted score results, and then select the top 20 microorganisms and evaluate the prediction performance of MADGAN through literature retrieval.

Asthma is a disease with heterogeneous process, accompanied by recurrent wheezing, chest tightness, dyspnea, indirect cough and other symptoms(Al-Moamary et al., 2021). It is reported that in 2010, about 8% of people were affected by asthma, especially in children, and the incidence rate is still rising(Guilbert et al., 2014). Asthma has been proved to be closely related to microorganisms(Çalışkan et al., 2013). For example, Haemophilia, Neisseria and Moraxella in the lungs of asthmatic patients have been proved to be closely related to the increased risk of neonatal oral and pharyngeal asthma, and Staphylococcus has been found in the respiratory tract of asthmatic children(Sullivan et al., 2016). These findings may provide a new method for the treatment of asthma. We choose the top 20 microorganisms related to asthma predicted by our model and then search the literature for further verification. The results are shown in the Table 3.

**Table 3:** The top 20 asthma-associated microbes predicted by MADGAN.

Rank	Microbe	Evidence
1	*Clostridium innocuum*	PMID:18672296
2	*Staphylococcus epidermidis*	PMID:6694502
3	*Streptobacillus*	PMID:6326694
4	*Burkholderiales bacterium* Smarlab 3,302,047	Unconfirmed
5	*Dorea*	PMID:30937143
6	*Stenotrophomonas maltophilia*	PMID:20537287
7	*Mannheimia*	PMID:10967288
8	Rikenellaceae	PMID:33204702
9	*Streptococcus parasanguinis*	PMID:17950502
10	*Yersinia*	PMID:10719781
11	*Alistipes*	PMID:33759390
12	*Corynebacterium*	PMID:22994424
13	*Erysipelotrichales*	PMID:22994424
14	*Mobiluncus*	Unconfirmed
15	*Cronobacter*	Unconfirmed
17	*Eubacteriaceae*	Unconfirmed
18	Unidentified bacterium ZF3	Unconfirmed
19	*Prevotellaceae*	PMID: 34422359
20	*Oxalobacteraceae*	PMID: 21194740

COPD is a lung disease that worsens over time, as long as the symptoms are shortness of breath and cough. By 2015, COPD patients accounted for about 2.4% of the global population (James et al., 2018). Due to the high smoking rate and aging population in developing countries, the death toll of COPD patients is rising rapidly. Although the treatment can delay the deterioration of COPD, there is no cure. Considering that there is a lot of evidence indicating the association between microbiome and COPD, for example, Galiana et al. (2014) found that the diversity of patients with high COPD was lower than that of patients with mild and moderate COPD. Therefore, we select the top 20 microorganisms related to COPD predicted by our model and then search the literature for further verification. The results are shown in the Table 4.

**Table 4:** The top 20 COPD-associated microbes predicted by MADGAN.

Rank	Microbe	Evidence
1	*Bacteroides*	PMID: 36498063
2	*Bacteroides* sp. CJ78	Unconfirmed
3	*Bacteroides vulgatus*	Unconfirmed
4	*Bacteroidetes*	PMID: 33063421
5	*Clostridiales bacterium* 80/3	Unconfirmed
6	*Clostridium cocleatum*	PMID:20857523
7	*Clostridium ramosum*	Unconfirmed
8	*Enterococcus*	PMID:24629344
9	*Erwinia*	Unconfirmed
10	*Escherichia*	PMID: 21605476
11	*Eubacteriaceae*	Unconfirmed
12	*Firmicutes*	PMID: 32353489
13	*Firmicutes bacterium* EG14	Unconfirmed
14	*Fusobacterium*	PMID: 35034433
15	*Verrucomicrobia*	PMID: 32295442
17	*Actinomyces*	PMID: 31174538
18	*Lachnospiraceae bacterium* A2	Unconfirmed
19	*Enterococcus faecalis*	PMID: 26623628
20	*Clostridia bacterium* TSW07CA7	Unconfirmeda

## 6. Conclusion

Deeply understanding the relationship between microorganisms and diseases can not only reveal the pathogenesis of more human diseases, but also provide new insights into disease prevention, diagnosis and treatment, thus promoting human health. Predicting the potential microbe-disease associations can help biologists to screen the most relevant microorganisms that cause diseases, thus reducing the time and cost of biological verification experiment (Zhou et al., 2017; Uchiyama et al., 2019). In this paper, we developed a deep learning model, named MADGAN, to predict potential microbe-disease associations. We adequately exploit multi-sources of abundant biological data to capture similarity features of microbes and diseases. This helps to predict new microbes (or new diseases) with few or no known association. In order to derive more informative representations, we propose graph convoluted neural network to learn representations for microbes and diseases. Meanwhile, the model is trained through the game between the generation network and the decision network. Finally, we utilized residual network and the cross-level weight distribution structure to enhance the depth of the network to prevent over-smoothing during model training. Comprehensive experiments demonstrated that MADGAN achieved satisfactory predictive performance.

However, although our model has good prediction performance, it still has some limitations and is expected to be further improved in the future. On the one hand, our model is a supervised learning framework, which means that our model cannot predict all new microorganisms and diseases. In the future, we will consider integrating multiple prior biological information, such as microbe-drug disease association and drug-disease association, to develop an unsupervised learning framework. On the other hand, it is still a huge challenge for MADGAN to forecast on large-scale datasets. In the future, we will consider integrating the results of multiple datasets to build datasets, so as to improve the prediction performance of the model on large datasets.

## Data availability statement

The original contributions presented in the study are included in the article/Supplementary material, further inquiries can be directed to the corresponding authors.

## Author contributions

WH and XY produced the main ideas, and did the modeling, computation and analysis and also wrote the manuscript. LW and XZ provided supervision and effective scientific advice and related ideas, research design guidance, and added value to the article through editing and contributing completions. All authors contributed to the article and approved the submitted version.

## Funding

This work was partly sponsored by the Hunan Provincial Natural Science Foundation of China (No. 2022JJ50138), the National Natural Science Foundation of China (No. 62272064), the Key project of Changsha Science and technology Plan (No. KQ2203001), the Science and Technology Innovation Program of Hunan Province (No. 2016TP1020), and the Hunan Provincial Education Department Scientific Research Project (No.20B080).

## Conflict of interest

The authors declare that the research was conducted in the absence of any commercial or financial relationships that could be construed as a potential conflict of interest.

## Publisher’s note

All claims expressed in this article are solely those of the authors and do not necessarily represent those of their affiliated organizations, or those of the publisher, the editors and the reviewers. Any product that may be evaluated in this article, or claim that may be made by its manufacturer, is not guaranteed or endorsed by the publisher.

## Footnotes

^1^ http://www.cuilab.cn/hmdad

^2^ https://disbiome.ugent.be/home

^3^ https://www.inetbio.org/humannet
